# Supplementary material for: A molecular toolbox to modulate gene expression and protein secretion in the bacterial predator Bdellovibrio bacteriovorus
Source: PLoS Genet. 2025 Nov 10;21(11):e1011935. doi: 10.1371/journal.pgen.1011935 (PMC12622784; doi:10.1371/journal.pgen.1011935)
Supplement: S1 Fig — (PDF) [file pgen.1011935.s001.pdf]

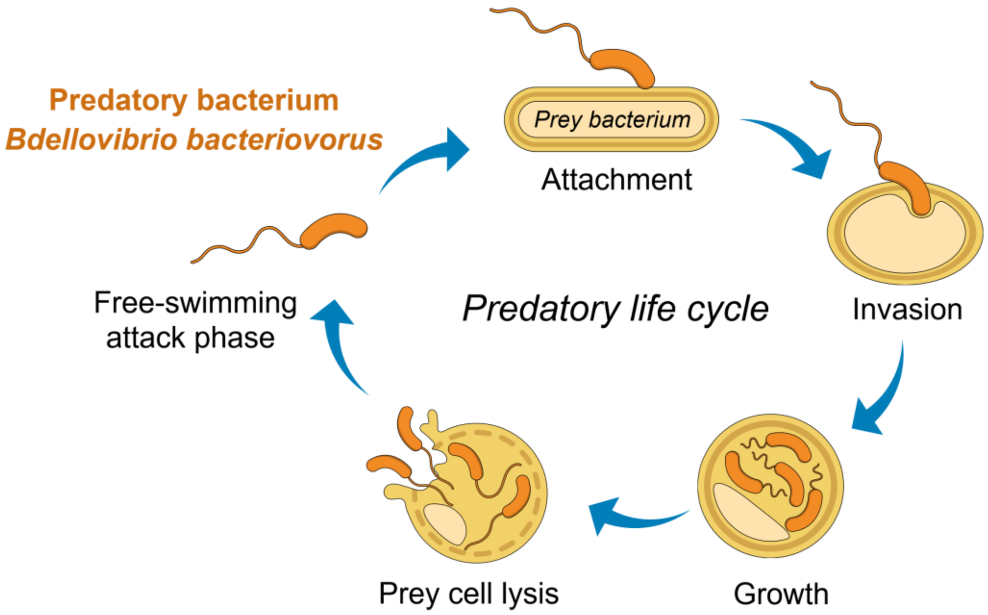

S1 Figure. Overview of the *B. bacteriovorus* predatory life cycle. During the free-swimming attack phase (AP), *B. bacteriovorus* swims towards and attaches to a Gram-negative bacterium (e.g. *Escherichia coli*). The predator then invades and multiplies within the prey periplasm, finally lysing the prey cell and releasing progeny back into the environment. These newly formed predator cells resume the attack phase, continuing their search for new prey bacteria. This image was assembled in the Mind the Graph platform (https://mindthegraph.com/) by Simona Huwiler and Ljiljana Mihajlovic. The latter two, as subscribed users, are entitled to full rights to their creation and are recognized as authors.
