# Supplementary material for: A molecular toolbox to modulate gene expression and protein secretion in the bacterial predator Bdellovibrio bacteriovorus
Source: PLoS Genet. 2025 Nov 10;21(11):e1011935. doi: 10.1371/journal.pgen.1011935 (PMC12622784; doi:10.1371/journal.pgen.1011935)
Supplement: S2 Fig — (PDF) [file pgen.1011935.s002.pdf]

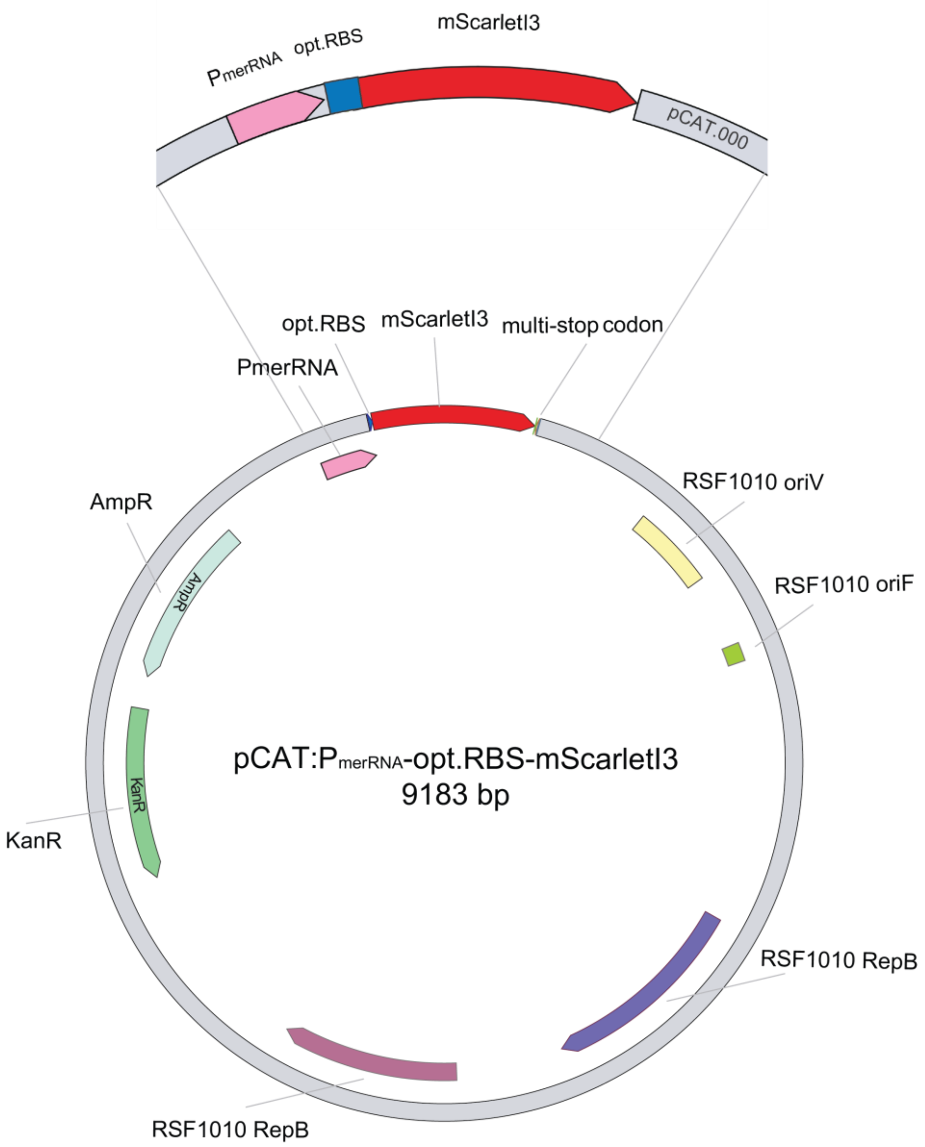

S2 Figure. Plasmid map of pLH-C1 (pCAT:P$_{merRNA}$-opt.RBS-mScarletI3), derived from pCAT.000 [1], showing the mScarletI3 reporter gene under the control of promoter P$_{merRNA}$ and an RBS sequence optimized for *B. bacteriovorus* (opt.RBS) [2]. Key features include kanamycin and ampicillin resistance cassettes (KanR, AmpR) for selection, as well as the RSF1010 origin of replication.

References:

1. Vasudevan R, Gale GAR, Schiavon AA, Puzorjov A, Malin J, Gillespie MD, et al. CyanoGate: A Modular Cloning Suite for Engineering Cyanobacteria Based on the Plant MoClo Syntax. Plant Physiol. 2019;180: 39–55. doi:10.1104/pp.18.01401

2. Dwidar M, Yokobayashi Y. Controlling *Bdellovibrio bacteriovorus* Gene Expression and Predation Using Synthetic Riboswitches. ACS Synth Biol. 2017;6: 2035–2041. doi:10.1021/acssynbio.7b00171
