# Supplementary material for: A molecular toolbox to modulate gene expression and protein secretion in the bacterial predator Bdellovibrio bacteriovorus
Source: PLoS Genet. 2025 Nov 10;21(11):e1011935. doi: 10.1371/journal.pgen.1011935 (PMC12622784; doi:10.1371/journal.pgen.1011935)
Supplement: S3 Fig — (PDF) [file pgen.1011935.s003.pdf]

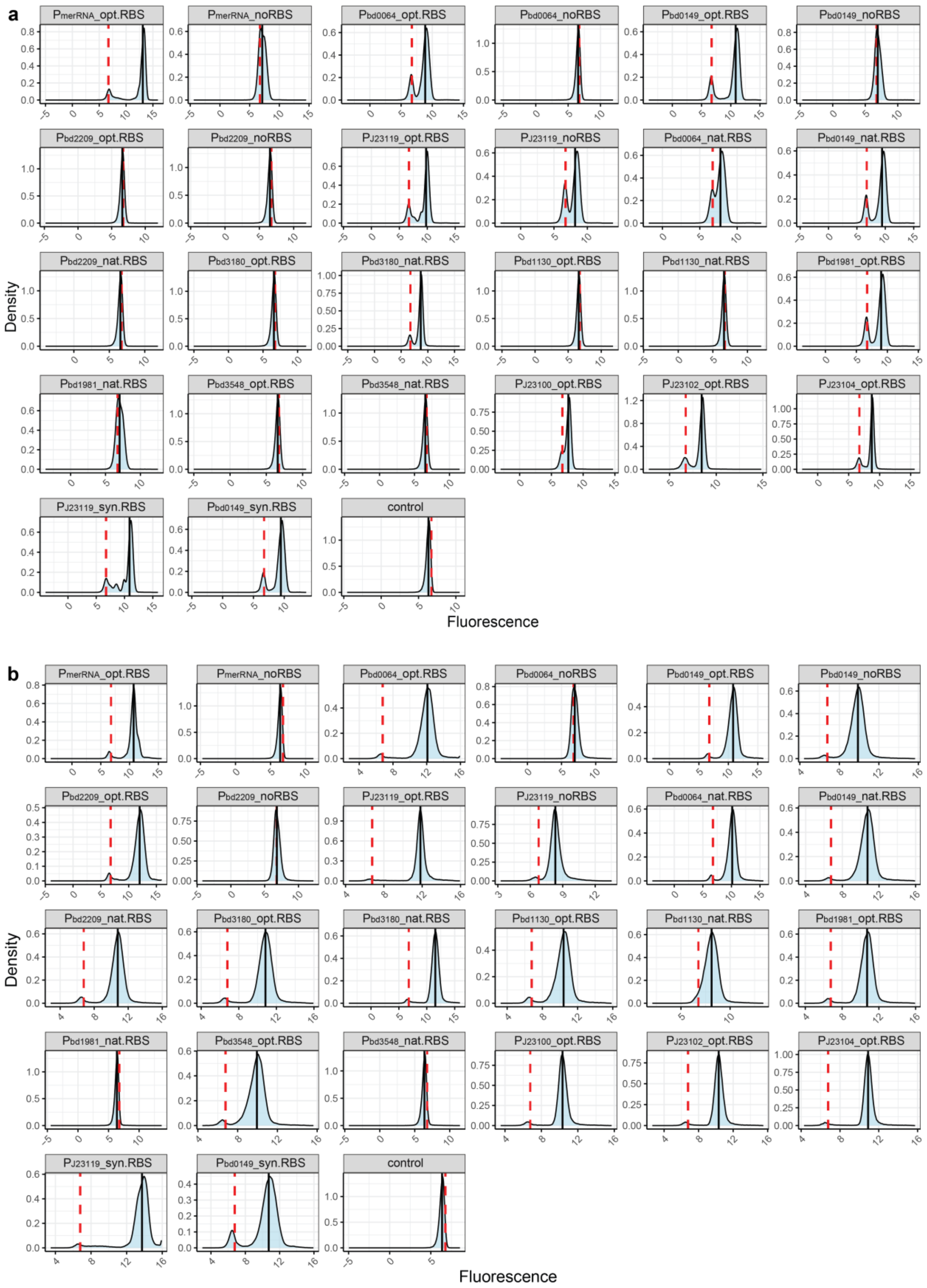

S3 Figure. Overview of mScarlet fluorescence distributions in cells harbouring various pCAT.000-derived vectors in *B. bacteriovorus* AP (a) and *E. coli* S17-1 (b). Thresholds (red, dashed line) indicate the 95th percentile of fluorescence intensity of the empty vector without reporter gene pCAT:P$_{merRNA}$-opt.RBS. Median fluorescence values are indicated by black lines. Panels show comparisons across different native and synthetic promoters combined with native (nat.RBS), optimized (opt.RBS), synthetic (syn.RBS), or no RBS (noRBS). Detailed plasmid description can be found in S5 Table.
