# Supplementary material for: A molecular toolbox to modulate gene expression and protein secretion in the bacterial predator Bdellovibrio bacteriovorus
Source: PLoS Genet. 2025 Nov 10;21(11):e1011935. doi: 10.1371/journal.pgen.1011935 (PMC12622784; doi:10.1371/journal.pgen.1011935)
Supplement: S4 Fig — (PDF) [file pgen.1011935.s004.pdf]

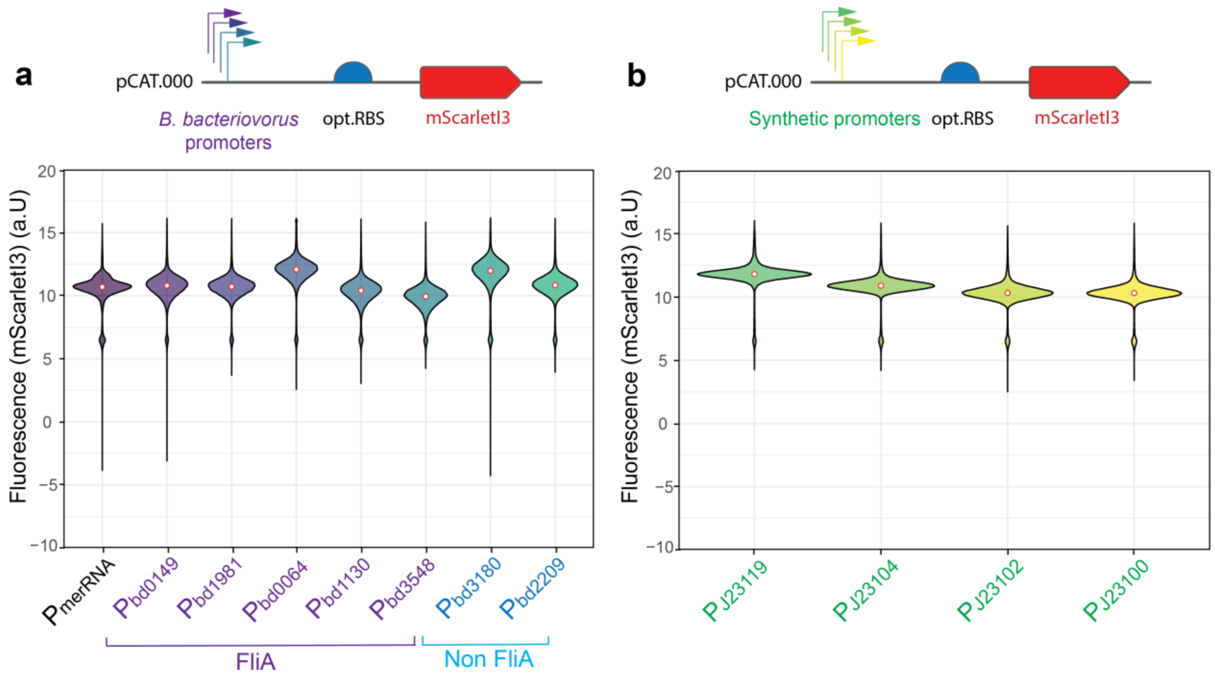


**S4 Figure. Native and synthetic promoters exhibit distinct mScarletI3 expression levels and variability in *E. coli* S17-1.** (**a**) Native *B. bacteriovorus* promoters, five with FliA motif ($P_{bd0149}$, $P_{bd1981}$, $P_{bd0064}$, $P_{bd1130}$, $P_{bd3548}$) and two without ($P_{bd3180}$, $P_{bd2209}$) were cloned upstream of mScarletI3 and introduced into *E. coli* S17-1. (**b**) Synthetic Anderson promoters ($P_{J23119}$, $P_{J23104}$, $P_{J23102}$, $P_{J23100}$) originally designed for *E. coli* drove varying levels of mScarletI3 expression under these same conditions. Each promoter was tested in a pCAT.000-derived plasmid with an optimized RBS upstream of mScarletI3. Fluorescence data was measured by flow cytometry. White dots in violin plots represent median fluorescence (density plots shown in S3b Fig). A second independent biological replicate yielded comparable results (see source data).
