# Supplementary material for: A molecular toolbox to modulate gene expression and protein secretion in the bacterial predator Bdellovibrio bacteriovorus
Source: PLoS Genet. 2025 Nov 10;21(11):e1011935. doi: 10.1371/journal.pgen.1011935 (PMC12622784; doi:10.1371/journal.pgen.1011935)
Supplement: S5 Fig — (PDF) [file pgen.1011935.s005.pdf]

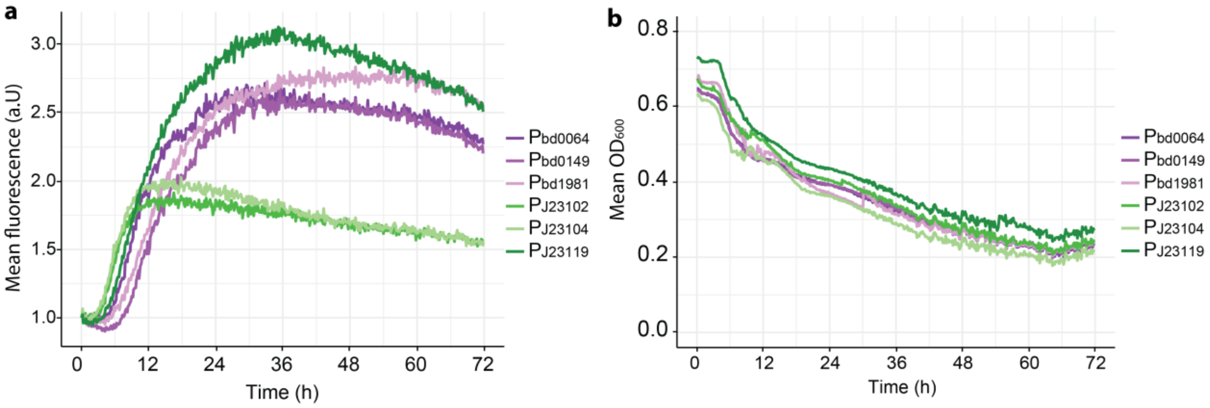

S5 Figure. Extended temporal gene expression of selected synthetic and native promoters in *B. bacteriovorus* populations during predation over 72 hours. (**a**) Overall gene expression level measured as mScarletI3 fluorescence at emission wavelength 590 nm during predation of *E. coli* S17-1 by *B. bacteriovorus* with native (P_{bd0064}, P_{bd0149}, P_{bd1981}) or synthetic ($P_{J23102,}$ $P_{J23104,}$ P_{J23119}) promoters expressed on pCAT.000-derived plasmids. For clarity of comparison, fluorescence starting values were adjusted to a common baseline across all samples. (**b**) Corresponding OD_{600} changes over the extended predation period. Data represents the average of two biological replicates, each measured in two technical replicates.
