# Supplementary material for: A molecular toolbox to modulate gene expression and protein secretion in the bacterial predator Bdellovibrio bacteriovorus
Source: PLoS Genet. 2025 Nov 10;21(11):e1011935. doi: 10.1371/journal.pgen.1011935 (PMC12622784; doi:10.1371/journal.pgen.1011935)
Supplement: S6 Fig — (PDF) [file pgen.1011935.s006.pdf]

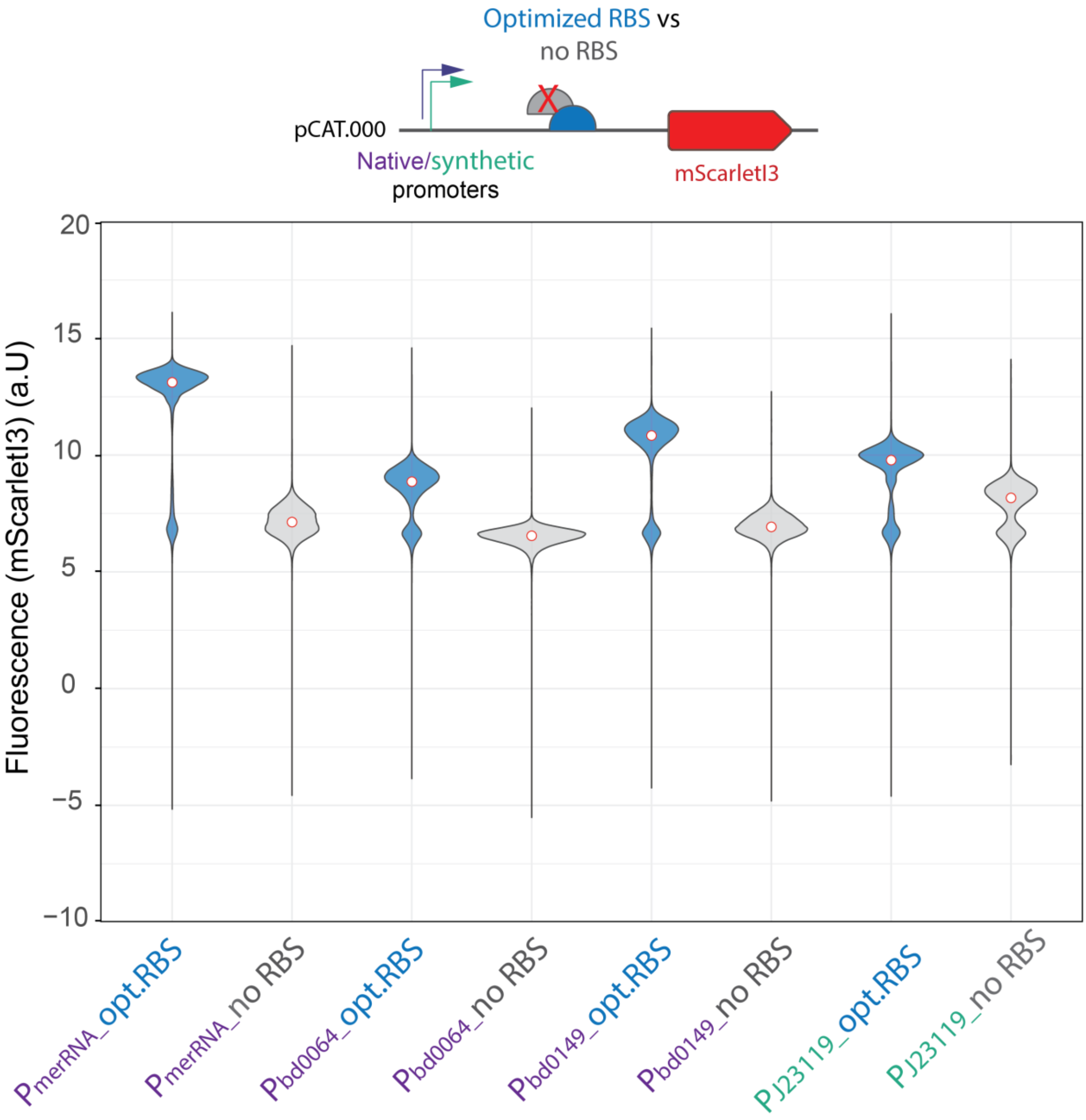

S6 Figure. Removal of Ribosomal binding site (RBS) from *B. bacteriovorus* native promoters and synthetic promoters leads to low expression level of mScarletI3 in *B. bacteriovorus* AP populations. Promoter strengths of *B. bacteriovorus* native promoters (P_{merRNA}, P_{bd0064}, P_{bd0149}) and the synthetic promoter P_{J23119} were assessed at the population level in *B. bacteriovorus* AP using mScarlet-I3 fluorescence measured by flow cytometry. For all promoters, the *B. bacteriovorus* optimized ribosome binding site (opt. RBS, blue) was compared to the same construct lacking an RBS (no RBS, grey). White dots in violin plots represent median fluorescence (density plots shown in S3a Fig). A second biological and independent repeat of these measurements showed the same outcome (see source data).
