# Supplementary material for: A molecular toolbox to modulate gene expression and protein secretion in the bacterial predator Bdellovibrio bacteriovorus
Source: PLoS Genet. 2025 Nov 10;21(11):e1011935. doi: 10.1371/journal.pgen.1011935 (PMC12622784; doi:10.1371/journal.pgen.1011935)
Supplement: S7 Fig — (PDF) [file pgen.1011935.s007.pdf]

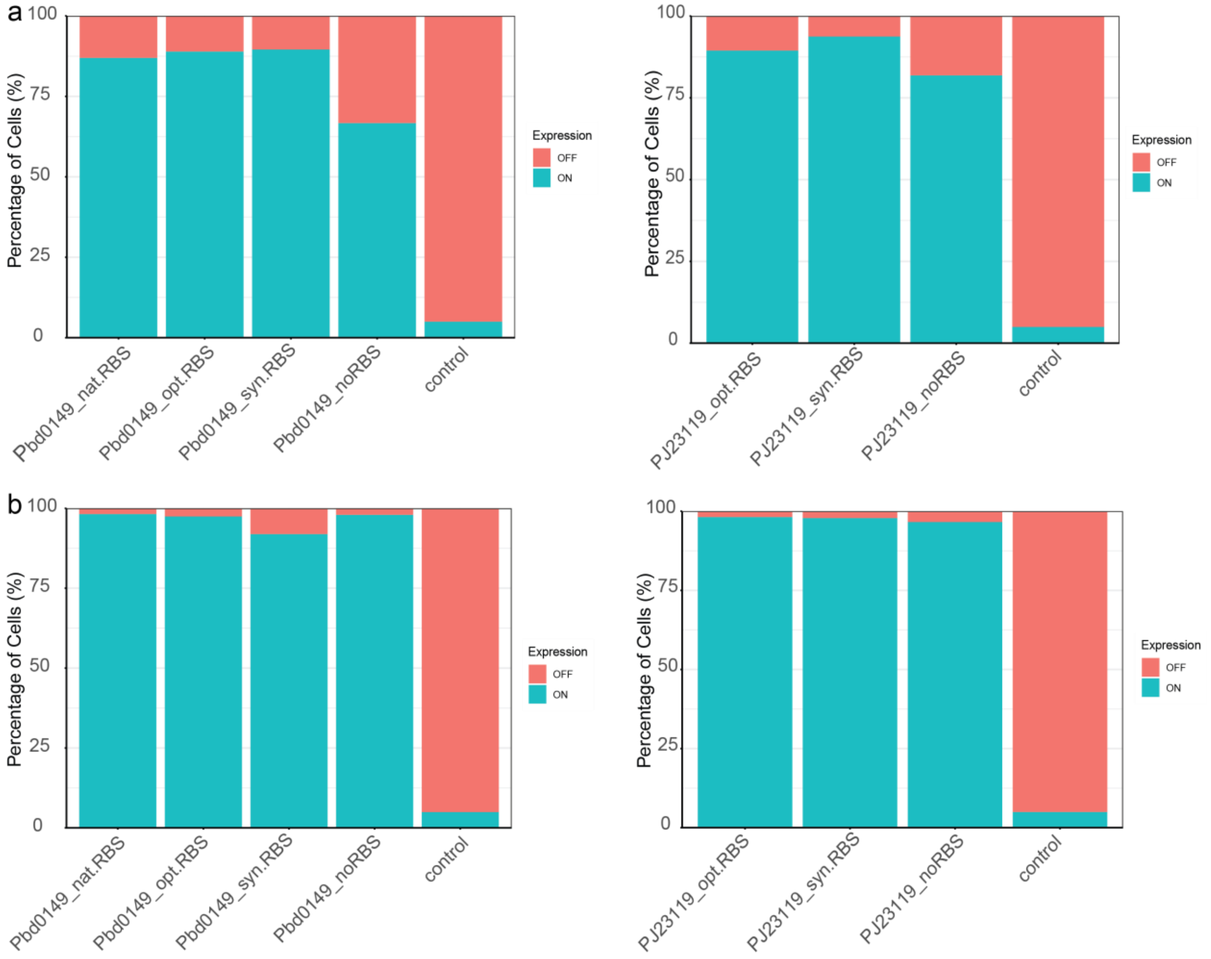

S7 Figure. Effect of promoter and ribosome binding site (RBS) on gene expression levels in *B. bacteriovorus* AP (a) and *E. coli* S17-1 (b). Bar plots represent the percentage of cells (%) exhibiting ON (cyan) or OFF (red) expression states, assessed using a fluorescent reporter gene of mScarletI3 on a pCAT.000-derived vector. Different combinations of the native promoter P_{bd0149} (left) and the synthetic promoter P_{J23119} (right) were tested with native (nat. RBS), optimized (opt. RBS), synthetic (syn. RBS), or no RBS (no RBS). Control represents cells harbouring empty vector without the reporter gene (pCAT:P_{merRNA}-opt.RBS). In general, the proportion of cells in ON state is larger in *E. coli* when RBS is absent compared to *B.bacteriovorus* AP (Figure S3), with exception of PmerRNA. Flow cytometry data was analysed as indicated in S7 Figure, where expression states were determined by gating cells based on fluorescence intensity thresholds set at the 95th percentile of the control. Most of this data is also shown as violin plots in Fig 4 and S4, S6 Figs.
