# Supplementary material for: A molecular toolbox to modulate gene expression and protein secretion in the bacterial predator Bdellovibrio bacteriovorus
Source: PLoS Genet. 2025 Nov 10;21(11):e1011935. doi: 10.1371/journal.pgen.1011935 (PMC12622784; doi:10.1371/journal.pgen.1011935)
Supplement: S1 Table — (PDF) [file pgen.1011935.s008.pdf]

S1 Table. Overview of promoter regions used in this study. Native promoter regions (violet stands for FliA regulated and light blue for non-FliA regulated, based on Karunker et *al*. [3]), native sequence containing native RBS (grey underlined), optimized RBS (dark blue, underlined) with additional AAG as space holder (blue) to translational start site, synthetic RBS BBa_B0034 (orange). Abbreviations: not applicable (n/a).

Native promoters	RBS	DNA Sequence 5`-> 3`	FliA regulation	Controlled gene
P_{merRNA}	opt.RBS	AAAGAAACCTGTAACAGGTTTCTTTTTTCATCCAATTTCTAATTTTCAATTTCAAAAAGCCCCTCATCGAGGGGCTTTTTCTGTTTCAAGTCTGAACAAATATCCGTCTGTTTTCCCACGCTGATACGCAAATCTGACGTAATTTGAGAATCGCACGTGGTC CCCTTGAAAATAAAACTTTGGTCGAGCTATCCTGATTTTGTTGAAACCAGACTTAAGGAGGCAACAAGATG	no	merRNA
P_{bd0149}	opt.RBS	GCGTCTCATTCTAATACAAGACCTAAAAACTTAAGAAACTCTGACTTTTGTCCGAAAAGGATATGGTAGGGGAAAGACTTAAGGAGGCAACAAGATG	yes	hypothetical protein
P_{bd0149}	nat.RBS	GCGTCTCATTCTAATACAAGACCTAAAAACTTAAGAAACTCTGACTTTTGTCCGAAAAGGATATGGTAGGGGAAATTTTATG	yes	hypothetical protein
P_{bd0149}	syn.RBS	GCGTCTCATTCTAATACAAGACCTAAAAACTTAAGAAACTCTGACTTTTGTCCGAAAAGGATATGGTAGGGGAAATCTAGAGAAAGAGGAGAAATACTAGATG	yes	hypothetical protein
P_{bd1981}	opt.RBS	GGGACGGGGAAAATGTCTCAAAACTGGACCTGATGAAAAAACTCGTTTAAGCGTCCCTGCACCTCATTTATTCTATAAAGGTGGGGGAGCTGACTTAAGGAGGCAACAAGATG	yes	hypothetical protein
P_{bd1981}	nat.RBS	GGGACGGGGAAAATGTCTCAAAACTGGACCTGATGAAAAAACTCGTTTAAGCGTCCCTGCACCTCATTTATTCTATAAAGGTGGGGGAGCTCATG	yes	hypothetical protein
P_{bd0064}	opt.RBS	TTTTCAGAATTTTAATCCGAAAAGTTAAAAATCACCCGGACCCCCTATTAATTTTTGGTAAGTAATACCGAAAGGATAGTATCCAATTTAAGACTTAAGGAGGCAACAAGATG	yes	hypothetical protein
P_{bd0064}	nat.RBS	TTTTCAGAATTTTAATCCGAAAAGTTAAAAATCACCCGGACCCCCTATTAATTTTTGGTAAGTAATACCGAAAGGATAGTATCCAATTTAAGCACAAGGAGTCTGACATG	yes	hypothetical protein
P_{bd1130}	opt.RBS	CCTAAGAAAGAGACTCACATATTAAGGATTCTTCATCGTGAAAAACTCGTTTTTTCGGTCAAAAAACCGATAAGAAAGACATGAGAAACGGGACTTAAGGAGGCAACAAGATG	yes	putative YapH protein
P_{bd1130}	nat.RBS	CCTAAGAAAGAGACTCACATATTAAGGATTCTTCATCGTGAAAAACTCGTTTTTTCGGTCAAAAAACCGATAAGAAAGACATGAGAAACGGAACGTCGCTTGTGTCATTCTTAGCACTCCTTTTTATG	yes	putative YapH protein
P_{bd3548}	opt.RBS	CTTTTGGGATCTCGTGGAATATCACAACTTCCCGCCGGACAGCTCTTTACGATTCTTTGATGGAGTCCGAAGAGTCCACCAGACTTAAGGAGGCAACAAGATG	yes	hypothetical protein
P_{bd3548}	nat.RBS	CTTTTGGGATCTCGTGGAATATCACAACTTCCCGCCGGACAGCTCTTTACGATTCTTTGATGGAGTCCGAAGAGTCCACCAATG	yes	hypothetical protein
P_{bd3180}	opt.RBS	TACCGACACGCTGAAGTCGCTCCATTTCGTCTGGACCAAAGATGGTCCCGCCTTTTGTCCAAAAGGTGTAGAATGGGTGTGGCTCATAGGTGACTTAAGGAGGCAACAAGATG	no	hypothetical protein
P_{bd3180}	nat.RBS	TACCGACACGCTGAAGTCGCTCCATTTCGTCTGGACCAAAGATGGTCCCGCCTTTTGTCCAAAAGGTGTAGAATGGGTGTGGCTCATAGGTGTTGAAATAGTGAGTGTTGTATAGAATGTGTTAAATCAAATTGAAAGATCAGGAGGATCACTATG	no	hypothetical protein
P_{bd2209}	opt.RBS	CTGGCGGACCGGGTGGAGGAGTTAAGCCTTCGCCGCGACTCGATTCTGAAAAGAATGATTTATTTTAAAGTTAAGAAAGTTAATACAACGAGACTTAAGGAGGCAACAAGATG	no	hemolysin-type calcium binding protein
P_{bd2209}	nat.RBS	CTGGCGGACCGGGTGGAGGAGTTAAGCCTTCGCCGCGACTCGATTCTGAAAAGAATGATTTATTTTAAAGTTAAGAAAGTTAATACAACGACGACGGCAAGTCTGTAGTTGGTGTTTGGGGGAAATTATG	no	hemolysin-type calcium binding protein
Synthetic promoters				
P_{J23119}	opt.RBS	TTGACAGCTAGCTCAGTCCTAGGTATAATGCTAGCGACTTAAGGAGGCAACAAGATG	no	n/a
P_{J23119}	syn.RBS	TTGACAGCTAGCTCAGTCCTAGGTATAATGCTAGCGACTTCTAGAGAAAGAGGAGAAATACTAGAAGATG	no	n/a
P_{J23104}	opt.RBS	TTGACAGCTAGCTCAGTCCTAGGTATTGTGCTAGCGACTTAAGGAGGCAACAAGATG	no	n/a
P_{J23102}	opt.RBS	TTGACAGCTAGCTCAGTCCTAGGTACTGTGCTAGCGACTTAAGGAGGCAACAAGATG	no	n/a
P_{J23100}	opt.RBS	TTGACGGCTAGCTCAGTCCTAGGTACAGTGCTAGCGACTTAAGGAGGCAACAAGATG	no	n/a

Reference:

3. Karunker I, Rotem O, Dori-Bachash M, Jurkevitch E, Sorek R. A Global Transcriptional Switch between the Attack and Growth Forms of *Bdellovibrio bacteriovorus*. Stevenson B, editor. PLoS ONE. 2013;8: e61850. doi:10.1371/journal.pone.0061850
