# Supplementary material for: A molecular toolbox to modulate gene expression and protein secretion in the bacterial predator Bdellovibrio bacteriovorus
Source: PLoS Genet. 2025 Nov 10;21(11):e1011935. doi: 10.1371/journal.pgen.1011935 (PMC12622784; doi:10.1371/journal.pgen.1011935)
Supplement: S2 Table — (PDF) [file pgen.1011935.s009.pdf]

S2 Table. Overview of the Amino acid and DNA sequences used for Sec-dependent signal peptides fused to NanoLuc reporter gene used in this study. Cleavage sites were predicted with SignalP 6.0 [4] and are indicated with a red slash.

N-terminal sec-dependent signal	Amino acid sequence	DNA Sequence 5`-> 3`
ss_Bd2269$_{1-22}$	MKFNVFALIVSVLFATSAQA/ER	ATGAAATTCAACGTGTTTGCACTCATCGTATCAGTGCTTTTCGCGACATCAGCTCAGGCAGAGCGC
ss_Bd0468$_{1-23}$	MKRPISLALSALTLTASLAHA/QE	ATGAAACGCCCTATTTCTTTGGCACTGTCCGCGCTGACTTTGACTGCTTCCCTGGCTCACGCCCAGGAG
ss_Bd0120$_{1-22}$	MKKTTLIAALILLSSAAAHA/GD	ATGAAAAAAACCACACTGATTGCAGCCTTGATCCTGCTAAGCTCTGCAGCTGCACACGCCGGTGAC
ss_Bd2692$_{1-20}$	MKRALLVGAVLFGSQAFA/GE	ATGAAACGTGCATTACTAGTTGGTGCGGTTCTGTTTGGTTCTCAGGCGTTCGCTGGCGAA

Reference:

4. Teufel F, Almagro Armenteros JJ, Johansen AR, Gíslason MH, Pihl SI, Tsirigos KD, et al. SignalP 6.0 predicts all five types of signal peptides using protein language models. Nat Biotechnol. 2022;40: 1023–1025. doi:10.1038/s41587-021-01156-3
