# Supplementary material for: A molecular toolbox to modulate gene expression and protein secretion in the bacterial predator Bdellovibrio bacteriovorus
Source: PLoS Genet. 2025 Nov 10;21(11):e1011935. doi: 10.1371/journal.pgen.1011935 (PMC12622784; doi:10.1371/journal.pgen.1011935)
Supplement: Attachment — Submitted filename: Response to Reviewers.pdf [file pgen.1011935.s015.pdf]

Reply from the authors

We would like to thank all reviewers for their comments on our manuscript. We reply to each comment in a **numbered response colored in blue**. Please be aware that we refer to the line number of the document 'Revised Manuscript with Track Changes'.

All data of the work presented in this manuscript including the data underlying the figures and results of this manuscript will **be available upon publication on Figshare** with doi 10.6084/m9.figshare.29565092. This will allow to implement changes if necessary. Figshare was chosen as a suitable repository, due to the big data amount produced by the flow cytometry raw data as the Zip-file folder is 4.85 GB big. The data is presented in a structured way in subfolders and an accompanying overview xls spreadsheet providing all the details on the raw data, R scripts used and second biological replicate datasets. Until this manuscript is accepted, this data can be accessed via private link (https://figshare.com/s/625ee38c7e1048646b0b) by the reviewers and the editorial team.

Reviewers' comments:

Reviewer #1:

One focus of microbiology is on predatory bacteria in recent years. A limited factor is a scarcity of toolkits for genetic operation. In this manuscript showed that authors developed a molecular toolbox for gene expression and protein secretion. To my knowledge, it is the second system for gene expression and the first system for protein secretion to Bdellovibrionota's bacteria. Authors showed a rational design, fine implementation for experiments, good english writing, and clear, understandable figures and tables. I appreciate this manuscript.

Response 1.0: Thank you very much for this positive feedback and your input on our manuscript.

Here I give one suggestion for the Title. This article is to focus on the development of a genetic toolkit for predatory bacteria, which is used for gene expression and protein secretion. Thus, my suggestion is to modify the title "Modulating gene expression and protein secretion in the bacterial predator Bdellovibrio bacteriovorus" as "A molecular toolbox development to modulate gene expression and protein secretion in the bacterial predator Bdellovibrio bacteriovorus".

Response 1.1: The authors agree to implement the "molecular toolbox" into the title. We adjusted the title to read "A molecular toolbox to modulate gene expression and protein secretion in the bacterial predator *Bdellovibrio bacteriovorus*" in the revised versions.

Two typewriting errors:

In Line 87, "…as in *E. coli*, are limited [12,28] To date," modified to "…as in *E. coli*, are limited [12,28]. To date,"

Response 1.2: Thank you for spotting the missing full stop. This was corrected (line 91).

In Line 89, “The scarcity of versatile gene expression systems than can be fine-tuned…”,

modified to “The scarcity of versatile gene expression systems that can be fine-tuned…”

Response 1.3: Thank you for detecting this typing error. In order to highlight the novelty of our work, this sentence was replaced in the revised form of the manuscript (lines 95-101).

Reviewer #2:

Major general comments:

The experimental work presented in PGENETICS-D-25-00685 is well done, the manuscript is clear and well written, and the topic is interesting. However, I have serious doubts about the novelty and impact of the work when considering the article published in 2024 by Salgado et al., entitled “Controlling the expression of heterologous genes in Bdellovibrio bacteriovorus using synthetic biology strategies” Microbial Biotechnology, 17, e14517.https://doi.org/10.1111/1751-7915.14517. This article was cited as reference # 28 in PGENETICS-D-25-00685, exclusively in the introduction section, as one of the few previous works describing a limited number of independent replicative plasmids and chemically inducible functional promoters. However, many of the aspects described in PGENETICS-D-25-00685, and claimed to be original work, had already been developed by Salgado et al. and this work were not properly cited, significantly diminishing the novelty and importance of Mihajlovic et al.'s work. The details of these aspects are provided below as “Specific detailed comments”.

Some aspects of PGENETICS-D-25-00685 can be considered original, such as the comparison of native versus synthetic promoters and the analysis of secretion systems in the predator. These aspects could be further developed.

Response 2.0: Thank you very much for your feedback on our manuscript. We fully agree with the reviewer that the novelty of our work lies in the comparison of native versus synthetic promoters, and the different secretion levels of a luciferase based on different secretion signals and promoters. While the latter secretion assays have been performed for the first time in *B. bacteriovorus* to the best of our current knowledge, this manuscript also enquires the impact of the ribosomal binding site on gene expression, which was not performed in Salgado et al., 2024. We consider it a particular strength of our manuscript that the same plasmid was used to measure both intracellular reporter protein production in *B. bacteriovorus* and reporter protein secretion. This design allows for a direct comparison of how differences in promoter strength affect secretion during protein expression. We fully agree that the work by Salgado et al. is highly relevant in the context of our manuscript, and we now more clearly highlight this work, as well as the additional aspects of our study, at several positions in the manuscript. The specific changes are listed below. The specific changes made in manuscript PGENETICS-D-25-00685 are addressed directly in the following sections, where the different discussion points are raised. Further, the introduction (line 90-102), abstract (lines 27-34) and author summary was adjusted (lines 46-55).

During the revision of this manuscript, another paper was published concerning inducible promoters and CRISPRi in *B. bacteriovorus* (de Pierpont et al. 2025, published 1st Sept 2025, DOI: 10.1093/femsml/uqaf021), which was submitted to the respective journal some days after this manuscript was publicly available online as Preprint (received 20th May 2025 at microLife, our preprint was publicly available on 17th May 2025). As this new article tested another set of promoters in

B. bacteriovorus (as it is the case for Salgado et al. 2024), a reference to the paper from de Pierpont et al. was implemented in the current manuscript for completeness (line 91).

Specific detailed comments that limit the novelty and impact of the work or require proper citation:

1) Development of a set of molecular tools for B. bacteriovorus through systematic fine-tuning of gene expression based on synthetic biology approaches. Although the technology applied in the two studies differs (Gibson assembly in PGENETICS-D-25-00685 versus the hierarchical assembly cloning technique Golden Standard (GS) based on the Golden Gate (GG) modular cloning used in Salgado et al), the result is similar and this is not mentioned or cited.

Response 2.1: Thank you very much for pointing this out. We have implemented a comparison to Salgado et al. regarding the gene expression using synthetic Anderson promoters in the "Verification and Comparison" and discussion section (lines 404-410, 690-691, 706-707). We further clarified the use of different cloning technologies in the discussion section (lines 740-755). Importantly, while the general conclusion of fine-tuning gene expression is shared, our study differs in both experimental design and scope, demonstrating that promoter-driven modulation of secretion can be systematically evaluated in *B. bacteriovorus*. This highlights the novelty and added value of our approach beyond the technical similarity.

2) Concerning the destination vectors, both works used plasmids carrying the ori RSF1010. This is not mentioned. In addition, Salgado et al developed destination vectors that adapt SEVA plasmids with a transposon Tn7-mediated chromosomal insertion system to monitor gene expression.

Response 2.2: Thank you for this input. We integrated in the discussion section (lines 727-739) that both plasmids have an RSF1010 ori**.** We also added a section mentioning the Tn7-mediated chromosomal integration (lines 750-755), which provides a complementary approach for monitoring gene expression. While chromosomal integration offers long-term stability, our plasmid-based system is uniquely suited for rapid testing of promoter–reporter combinations and secretion assays. The advantages of both methods are described in lines 742-755.

3) Both studies developed a system based on fluorescent proteins to monitor gene expression by flow cytometry at the single-cell level. In this sense, Salgado et al. observed that predator cells carrying RSF1010 replicative vectors exhibited heterogeneity in terms of their fluorescent phenotype: some cells accumulated high levels of fluorescent proteins, while others accumulated lower or none levels. For this reason, these authors applied a monocopy chromosomal insertion system to monitor gene expression. Similar behaviour was observed in the results presented in Figures 1, 2, 4 and S3-S4, but this is not mentioned in the text.

Response 2.3: Thank you for bringing up the results on the RSF1010-based plasmid expression with pSEVA plasmids in Salgado et al. They show a heterogeneous expression of the fluorescent protein reporter based on pSEVA by microscopy (Salgado et al. Fig. 3, Fig S5). The latter is nicely confirmed in Salgado et al. Fig. S7 for most promoters, while PJ116 truly shows two distinct positive populations. While the pCAT-plasmids we used also have an RSF1010-derived ori as the pSEVA by Salgado et al., we mainly detected different expression levels based on a Gaussian distribution. The small subpopulation typically visible around 6 a.U. in Fig. 2 and Fig. 4 originates from the cut-off negative cell population (see S3 Fig.). The only case where we see different subpopulations of gene expression

levels in “on” state with the expressions above the threshold levels is the pCAT-derived plasmid with PJ23119 and the synthetic RBS or optimized RBS (Fig.4, S3 Fig). A potential difference of expression might have been caused by other differences in the plasmids like for example the local plasmid context and the type of selective antibiotics as in this study Kanamycin was used. This comparison was implemented and discussed in the discussion section (lines 727- 739).

4) Both works monitor gene expression in the predator using synthetic promoters from the Anderson Collection. In one case, they even use the same promoter (PJ23102 in PGENETICS-D-25-00685, named PJ102 in Salgado et al.). I notice a serious lack of novelty here.

Response 2.4: Thank you for this comment. We agree that both studies used Anderson synthetic promoters, and we have now acknowledged this overlap explicitly in lines 90-91, 404-410, 690-691, 706-708. Our work extends beyond the findings of Salgado et al. building systematically on four different Anderson promoters we exchanged and modified ribosome binding sites (RBSs) to systematically evaluate their effect on expression levels. Thus, while Salgado et al. demonstrated that synthetic promoters are functional in *B. bacteriovorus*, our study expands this approach by further expanding the promoter set and dissecting the contribution of RBS sequences to gene expression.

5) Regarding the discussion about the feasibility of expressing synthetic promoters in the predator as a consequence of the existence of the appropriated σ factors in B. bacteriovorus, it was addressed here and also before in Salgado et al, but not mentioned in PGENETICS-D-25-00685.

Response 2.5: Thank you for bringing this to our attention. We have now explicitly mentioned in the discussion that the functionality of synthetic promoters in *B. bacteriovorus* is linked to the presence of the highly similar σ factors, as previously mentioned by Salgado et al. (line 706-712)This addition provides context and acknowledges prior discussion of this mechanism, while highlighting that our study independently confirms and extends these observations.

Other comments:

6) The comparison between synthetic and native promoters is interesting and should be developed further, but highlighting previous works and describing the novel aspects of the study.

Response 2.6: We highlighted previous work by extending the introduction to mention and cite previous (& very recent) studies of promoters (line 89-98) as well as in lines 404-410, 706-712). The novelty of the manuscript investigating the influence of the RBS and the fine-tuning of secretion is implemented in the introduction in line 95-98. The abstract and author summary were slightly adjusted to point out the novel aspects of the influence of RBS on gene expression, and most importantly the fine-tuning of protein secretion.

7) The section describing the evaluation of different ribosomal binding sites to fine-tune gene expression is interesting and novel, but a broader range of RBS must be considered to improve the significance of the results.

Response 2.7: We agree that a broader range of RBS variants would further strengthen the conclusions. However, we view our work as a solid starting point for this line of investigation. A more comprehensive analysis would require the generation and screening of RBS libraries, which is beyond the scope of the present study but represents a valuable direction for future work (mentioned in line 812-815).

8) I found the section describing the analysis of different native Sec-dependent signal sequences very original. Perhaps this section could be further developed to enhance the novelty and impact of the work.

Response 2.8:

We thank the reviewer for the positive evaluation and the suggestion. We expanded the discussion noting that our analysis of four different Sec-dependent signal sequences provides an initial framework for further studies (lines 774-776). In this study four different native Sec-dependent signal sequences were tested, one of which (Bd0468) was shown previously to be involved in secretion into the Bdelloplast by microscopy of Bd0468-mCherry (Lambert et al. 2016, Sci Rep) (now mentioned in line 641-643). We anticipate that future research on secretion by *B. bacteriovorus* will evaluate further specific Sec-dependent signal sequences of interest and advantage for specific future studies and purposes, as there is likely an effect of the secreted protein of interest structure on the efficiency of secretion and recognition of the N-terminal Sec-dependent signal sequence. Currently, we are starting to further develop this system by expressing different types of proteins to expand the repertoire. However, this would be beyond the scope of this methods paper. The novelty and impact of the work presented in this manuscript is in testing different RBS combinations with different promotors for internal protein expression, while at the same time using the same system to establish a novel secretion assay for a periplasmic predatory bacterium. The latter shows that the amount of reporter protein (NanoLuc) secretion can be regulated by using different signal sequences or promoters. This systematic approach also allows to compare the very same promoters regarding cell internal expression to secretion levels.

Reviewer #3:

The authors describe their contribution to the B. bacteriovorus genetic toolbox, including the systematic assessment of RBS's, synthetic and natural promoters and the development of a nanoluc-based secretion assay. Previously, several promoters were tested and introduced for B. bacteriovorus, but the toolbox is still very limited. This study is an addition to the field, especially as the authors used single-cell methods (FACS) to test their promoters behavior in attack phase. The study employs the nanoluc system in B. bacteriovorus for the first time to assess secretion and the efficacy of secretion tags. The study is carried out well and a welcome addition to the still limited B. bacteriovorus toolbox.

Response 3.0: Thank you very much for this positive assessment of our manuscript.

I have only few comments:

1) In the last years, several studies introduced synthetic and non-native promoters in B. bacteriovorus. It would have been a nice addition to the study to include these already used promoters (for instance Pbiofab (Kaljevic et al, 2021), PexD (Salgado et al, 2024)) as a benchmark/comparison.

Response 3.1: We acknowledge the suggestion of having a benchmark for gene expression in our study. We used natural promoter P_{bd0064} as a benchmark in our study, since Bd0064 -based fluorescent reporters (mCherry and others) have been used as one of the first in the field and in many different *B. bacteriovorus* studies (e.g. Willis et al., 2016 https://doi.org/10.1016/j.cub.2016.09.067, Findlay et al, 2019 https://doi.org/10.1038/s41598-019-43467, Tyson et al., 2024 https://doi.org/10.1038/s41467-024-47412-3). Based on our limited capacity, we did not include other benchmarks for comparison to non-native promoters in *B. bacteriovorus* (like Pbiofab (Kaljevic et al, 2021), PexD (Salgado et al, 2024)). We decided to focus on the Anderson library, which is widely used for the purpose of developing molecular toolbox in various different kinds of bacteria (e.g. Myxobacteria (Hu_2021), Cutibacterium (Nevot_2025)). We used Anderson promoter P J23119 as a benchmark, as P J23119 is commonly used as a benchmark in other promoter characterization studies. The latter is now mentioned in line 398-399 with according references.

2) It is notoriously difficult to do single-cell analysis on B. bacteriovorus during the growth phase, so it is understandable that the authors did not add heterogeneity studies to their promoter assessment in growth phase. However, would it not be possible to at least make a qualitative (and possibly also quantitative) assessment of the single cell behavior and promoter strength in GP using fluorescence microscopy?

Response 3.2: In our study, we focus on *B. bacteriovorus* in the free-swimming attack phase (AP) as this phase is of big importance for the use of *B. bacteriovorus* in different future applied settings. In addition, the natural promoters we chose are specifically expressed in the AP. We tried to achieve flow cytometry measurements during the GP of *B. bacteriovorus* within the Bdelloplast using a strain expressing mCherry. However, the signal was too weak for any quantification. Therefore, we focused only on AP in our study. We decided to use flow cytometry in our study and not microscopy, since the amount of single cell high throughput generated for flow cytometry is much larger, allowing a faster assessment of different expression levels over a whole bacterial population expressing a reporter gene. Further characterisation of promoters active during GP in combination with in-depth microscopy studies on the single-cell level will be of interest for other future studies. In fact, while this manuscript was under peer review (while already being publicly available on BioRxive since 17th May 2025), a new paper from the Laloux lab (de Pierpont et al, 2025, microLife, DOI: 10.1093/femsml/uqaf021, submitted to microLife on 20th May 2025) was published on 1st September 2025 addressing the issue of inducible promoters during GP in *B. bacteriovorus* with detailed microscopy studies at single-cell resolution. We now mention the Paper of de Pierpont in line 91.

Further small changes made by the authors to the manuscript since the original submission:

1) The Manuscript structure was adjusted to adhere to the standard Methods article layout. To accommodate the “Application” section better, the panels in Fig. 5b and Fig.5c and the according paragraphs were interchanged. Further, one promoter name was updated.

2) In S1 Figure the *Bdellovibrio bacteriovorus* icon in the scheme was replaced to show a dampened flagellum.

3) Fig. 2 was updated as a dot was missing in the legend description of the axis (a.U.).
